# Biofeedback for treatment of awake and sleep bruxism in adults: systematic review protocol

**DOI:** 10.1186/2046-4053-3-42

**Published:** 2014-05-02

**Authors:** Sasa Ilovar, Danaja Zolger, Eduardo Castrillon, Josip Car, Kit Huckvale

**Affiliations:** 1 Global eHealth Unit, Department of Primary Care and Public Health, School of Public Health, Imperial College London, 306 The Reynolds Building, St Dunstan's Road, London W6 8RP, UK; 2 Faculty of Medicine, University of Ljubljana, Vrazov trg 2, 1000 Ljubljana, Slovenia; 3 Section of Clinical Oral Physiology, School of Dentistry, Health, Aarhus University, Vennelyst Boulevard, 9 8000 Aarhus, Denmark

**Keywords:** Biofeedback, Awake bruxism, Sleep bruxism, Adults, Systematic review

## Abstract

**Background:**

Bruxism is a disorder of jaw-muscle activity characterised by repetitive clenching or grinding of the teeth which results in discomfort and damage to dentition. The two clinical manifestations of the condition (sleep and awake bruxism) are thought to have unrelated aetiologies but are palliated using similar techniques. The lack of a definitive treatment has prompted renewed interest in biofeedback, a behaviour change method that uses electronic detection to provide a stimulus whenever bruxism occurs. This systematic review aims to provide a comprehensive overview of the state of research into biofeedback for bruxism; to assess the efficacy and acceptability of biofeedback therapy in management of awake bruxism and, separately, sleep bruxism in adults; and to compare findings between the two variants.

**Methods:**

A systematic review of published literature examining biofeedback as an intervention directed at controlling primary bruxism in adults. We will search electronic databases and the grey literature using a predefined search strategy to identify randomised and non-randomised studies, technical reports and patents. Searches will not be restricted by language or date and will be expanded through contact with authors and experts, and by following up reference lists and citations. Two authors, working independently, will conduct screening of search results, study selection, data extraction and quality assessment and a third will resolve any disagreements. The primary outcomes of acceptability and effectiveness will be assessed using only randomised studies, segregated by bruxism subtype. A meta-analysis of these data will be conducted only if pre-defined conditions for quality and heterogeneity are met, otherwise the data will be summarized in narrative form. Data from non-randomised studies will be used to augment a narrative synthesis of the state of technical developments and any safety-related issues. PROSPERO registration number: CRD42013006880.

**Discussion:**

Biofeedback is not new, but its place in the clinical management of bruxism remains unclear. New research, and the availability of miniaturized consumer-grade devices, makes a systematic review timely to guide treatment decisions and inform future research.

## Background

### Description of the problem

Bruxism is a repetitive jaw-muscle activity characterised by clenching or grinding of the teeth and by bracing or thrusting of the mandible [1]. Bruxism may be either primary (idiopathic) or secondary [2,3]. Primary bruxism is divided into two types that are thought to be clinically unrelated: sleep and awake bruxism. Sleep bruxism (SB) is involuntary and is classified as a sleep-related movement disorder [1]. Awake bruxism (AB), by contrast, is defined as the awareness of jaw clenching, and appears to be semi-voluntary [2-4]. In adults under 65, the prevalence of SB is around 10% and declines gradually with age [2,3,5]. AB prevalence is probably higher and, unlike SB, is more frequently observed in females [4,5]. Secondary bruxism has been observed as a side effect of medication use and in some neurological and developmental disorders. In the future, it may be possible to further differentiate forms of bruxism according to the underlying cause and clinical manifestations [6].

Bruxism is considered to have a multifactorial aetiology that includes currently poorly defined aspects of central nervous system function, genetic and behavioural factors [2-4]. While AB is currently considered to be a parafunctional reaction to mental or physical stress [3,4], SB may be a pathological variant of normal physiological activity involved in upper airway patency maintenance and oesophageal lubrication [1,7]. Patients with SB exhibit patterns of sleep arousal not seen in usual controls or patients with AB [4,8,9]. However, stressors and mood also appear to play a role in SB [4], and both conditions display patterns of altered autonomic sympathetic activation [10] and neurochemistry [11]. A number of other conditions may coexist with bruxism, including temporomandibular joint disorder (TMD), orofacial pain, headaches and sleep-disordered breathing. The causal relationships between these conditions and bruxism remains unclear [8]. Some authors view bruxism as an underlying cause of TMD, while others consider both bruxism and TMD as related consequences of abnormal muscle activation. The management of bruxism is not affected by coexisting TMD.

Episodes of bruxism consist of masticatory muscle activity during wakefulness or sleep and manifest as a range of signs and symptoms in the orofacial region [12]. The symptoms of AB and SB are similar and include tooth grinding, jaw-muscle discomfort with or without frank pain [13], temporomandibular joint tenderness, facial pain and headache [8]. SB symptoms are usually worst in the morning on waking and improve during the day, while patients with AB may develop symptoms only after waking [4]. Signs include abnormal tooth wear, tongue indentation, increase in jaw muscle activity (measured by electromyograph (EMG) or polysomnograph (PSG)), masseter muscle hypertrophy, reduction in salivary flow, lip or cheek biting, gum recession, limitation of ability to open the mouth, and burning tongue [2,13,14]. The negative consequences of bruxism include subjective impacts like stress, anxiety, tiredness and poor sleep quality, as well as damage to teeth, which may ultimately result in premature loss of dentition [8].

Because of the variety of symptoms and overlap with other conditions, diagnosis of bruxism requires a careful process of assessment that incorporates questionnaires, history taking and examination. Objective testing is uncommon outside research settings, but includes EMG recording of the activity of the masticatory muscles and PSG recording of the sleeping patient. While full audio-video PSG recording remains the gold standard for diagnosis of SB [1,14,15], standardized clinical diagnostic criteria have also been proposed [1,15,16]. There are no validated objective tests for the diagnosis of AB, which relies instead on direct questions and visual observation of patient behaviour [3].

No therapy to date has been shown to effectively and permanently cure bruxism [2,8]. Current treatment focuses on symptom management and prevention of further complications [8,17,18]. The most widely accepted management approaches are intraoral appliances, pharmacotherapy (for SB), and behavioural strategies [8].

Intraoral appliances (occlusal splints and anterior tooth appliances) protect teeth from pathological wearing and relax the masticatory muscles. The palliative effect of intraoral appliances seems to be transient, however, and may result in side effects that include pain and, less commonly, altered occlusion that interferes with bite [8].

Pharmacological agents have been used to treat SB by targeting neurochemical systems involved in orofacial motor activity. Despite experimental use of a variety of compounds, including benzodiazepines and other muscle relaxants, antidepressants, anticonvulsants, dopaminergic drugs and beta blockers [2,8], there is currently limited evidence supporting routine application in clinical practice [19]. Although botulin toxin may have a future role [20], there are no widely accepted pharmacological approaches for the management of AB [1].

Characterisation of bruxism as a parafunctional activity rather than a muscle disorder *per se* has prompted interest in the scope for cognitive and psychological interventions that might modify the behaviour directly. Behavioural approaches that have been attempted include patient education, sleep hygiene, habit retraining, relaxation techniques, meditation, hypnotherapy, autosuggestion, psychoanalysis, cognitive behavioural therapy and biofeedback [8,17]. Conclusions concerning each of these have historically been limited by the number and quality of available studies [2,8,17].

Techniques directed specifically at pain relief include transcutaneous electrical nerve stimulation, acupuncture and manual massage. While potentially beneficial for the subset of patients where pain is a major management issue, there is a lack of robust evidence justifying clinical use [21].

### Description of the intervention

Biofeedback is a technique that provides individuals with information about their bodily functions with the intention of promoting changes in behaviour that result in improved health or performance [22,23]. Electronically detected physiological measurements are coupled with a feedback signal that is initiated when pre-specified criteria are met and terminated only when the desired change in behaviour occurs. Biofeedback aims to generate a learned response that persists even after the technique is discontinued [24]. Feedback can be provided in a number of ways; for example, as visual information displayed to a patient or as a sensory stimulus. Biofeedback has been employed for a range of conditions including urinary and faecal incontinence [2], essential hypertension [25], rehabilitation after stroke [26] and in maintenance of mobility and gait in older patients [27]. The most convincing evidence of the potential efficacy of biofeedback comes from studies examining feedback as an adjunct to muscle exercises for the treatment of female pelvic floor dysfunction [28].

**Figure 1:**
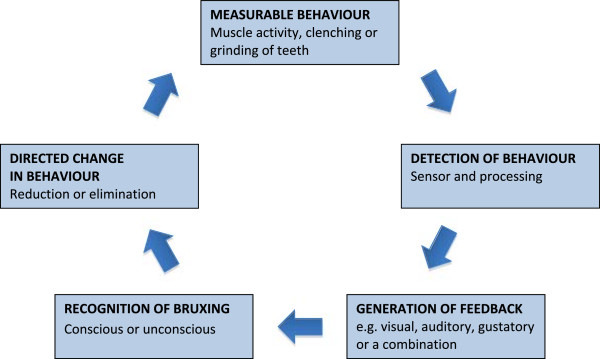
Schematic mode of action of biofeedback for bruxism.

Bruxism biofeedback typically involves a contemporary stimulus generated in response to a detected grinding or clenching event (Figure 1). Detection may rely on mechanical sensors integrated into splints, EMG or PSG analysis, with subsequent electronic analysis based on patterns of activity meeting pre-specified criteria. The paired feedback may be auditory, vibratory, electrical or gustatory and, in the case of SB, can either be intended to be waking or non-waking [29]. In wakeful patients, the stimulus is intended to promote awareness of clenching and prompt relaxation of the jaw muscles as well as permit reflection on the context or patterns of thought that might have given rise to the bruxism. The stimulus is not intended to be noxious but to be of sufficient magnitude to, at least initially, intrude into conscious thought and alert the patient. For sleeping patients, stimuli may either be intended to disrupt sleep or to provide a non-waking stimulus that is processed in some way by the sleeping brain. The recent finding that conditioned learning can occur during sleep in humans [30] suggests that biofeedback need not wake a patient to be effective. Because biofeedback is intended to cause a learned change in behaviour, there is the prospect of long-term reduction or elimination of symptoms in addition to palliating the condition by disrupting bruxism during the period of active treatment.

A related mode of management for SB is contingent electrical stimulation (CES), in which feedback is provided as an electrical stimulus applied to the skin, lip, or masticatory muscles. The possibility of local effects including changes in biochemistry in stimulated tissues, in addition to higher cognitive responses, sets CES apart from other forms of bruxism biofeedback.

### Adverse effects of the intervention

Adverse effects associated with biofeedback are most likely to centre on the feedback stimulus. Frequent arousal may lead to fatigue and, in SB, sleep disruption and consequent daytime sleepiness [3,4]. Sleep disturbance may affect bed partners. Some forms of biofeedback can include sensations which may be unpleasant and intended to cause an aversive response, for example by generating an unpleasant taste. When the stimulus is electrical, pain may be experienced if the magnitude is not configured appropriately. All adverse effects are expected to be transient and would cease if the intervention were discontinued.

### Why it is important to do this review

Interest in biofeedback management of bruxism has recently revived. Two factors appear to be relevant. Firstly, there remains no satisfactory definitive treatment for bruxism on grounds of efficacy and acceptability. Secondly, recent technical advances permit smaller and cheaper biofeedback appliances that may now make wider uptake feasible.

This review will complement a recent systematic review which failed to find strong evidence to support the use of biofeedback technology on SB treatment and highlighted the confusing diversity of different forms of biofeedback available [31]. In response to this, we will generate a taxonomy of biofeedback types and use this to structure our analysis. We will also include AB, recognising the potential to compare and contrast aspects of intervention design and findings between the two condition variants.

### Objectives of the review

We aim to: (1) provide a comprehensive overview of the state of research concerning biofeedback for bruxism, including a taxonomy of different types of biofeedback; (2) assess the efficacy and acceptability of biofeedback therapy in management of AB and, separately, SB in adults; and (3) characterise shared issues concerning the use of biofeedback for AB and SB as well as issues specific to each.

## Methods/design

### Review design

A systematic review combining an initial synthesis to describe the state of research and present a taxonomy of biofeedback types and, if appropriate, a subsequent meta-analysis of trial evidence for the efficacy of biofeedback for SB and, separately, AB. If no meta-analysis is appropriate then we will present a narrative summary of trial results. The conduct of the review will be based on the *Cochrane Handbook for Systematic Reviews of Interventions, Version 5.1.0*[32].

### Criteria for including studies in the review

#### ***Participants***

We will include studies of adults aged 18 years or older with either: a clinical diagnosis and/or ambulatory EMG/electrocardiograph/PSG diagnosis or full PSG diagnosis of SB; or a diagnosis of AB, identified by means of direct questions and visual observation of patient behaviour [3]. For SB, we will accept any diagnosis based on clinical or research PSG criteria that are consistent with the International Classification of Sleep Disorders Second Edition criteria (Table 1) [15]. We will include adults with coexisting diagnoses that may be related to bruxism but which do not affect clinical management (for example, TMD).

**Table 1:** Clinical and polysomnographic criteria for diagnosis of sleep bruxism

**Clinical criteria**	**Polysomnographic criteria**
The participant reports or is aware of tooth grinding or clenching during sleep (3–5 nights per week over past 6 months) and one or more of the following:	Polysomnographic monitoring demonstrates both of the following: jaw muscle activity during the sleep period and absence of associated epileptic activity.
	Polysomnographic diagnostic cut-off criteria:
- Abnormal tooth wear,	- More than four bruxism episodes per hour;
- Jaw muscle discomfort, fatigue or pain, and jaw lock upon awakening,	- More than six bruxism bursts per episode and/or 25 bruxism bursts per hour of sleep; and
- Masseter muscle hypertrophy evident on voluntary forceful clenching.	- At least two episodes with grinding sounds.

We will exclude participants with: known neurological and psychiatric comorbidities, secondary bruxism [33]; and/or a past history of receiving biofeedback for bruxism or co-existing alternate therapy for bruxism because this may affect how these patients will respond to treatment.

### Interventions

We will include studies that use biofeedback as an intervention directed at reducing or controlling bruxism. Qualifying interventions must pair a mechanism for detecting bruxism (for example, EMG analysis) with a contemporary feedback mechanism (for example, auditory warning). We will place no restriction on the nature of either the detection mechanism or the feedback, but will constitute subgroups to explore the relative effect of different types of monitoring and feedback. In particular, because the learning potential associated with CES remains unclear, we will analyse CES studies separately from other forms of biofeedback.

We will not include studies that use biofeedback to warn about elevated tension in mandibular musculature, rather than bruxing, since the mode of action of such interventions in preventing future bruxism is unclear and may not be the same as techniques that provide contemporary feedback about detected bruxism [34-37]. We will consider interventions of any duration (provided that duration is clearly defined).

### Comparisons

We will include comparisons with placebo (sham biofeedback) and other types of intervention for bruxism including behavioural strategies, oral appliances and pharmacological treatments. We will also include comparisons between different types of biofeedback.

### Outcomes

We will consider the following primary and secondary outcomes, which are defined in respect of review Objective 2.

### Primary outcome

1a. Frequency of bruxism episodes measured with either EMG or PSG (number of bruxing episodes per day/night/otherwise specified time frame).

1b. Duration of bruxism episodes measured with either EMG or PSG (number of time units per episode).

### Secondary outcomes

2. Self-reported (or reported by sleeping partners in SB) frequency of bruxism episodes (approximate number of times bruxing occurs during the day/night).

3. Self-reported pain (facial pain, myofascial headache) assessed using a validated instrument (points on different scales for subjective assessment of pain or questionnaires).

4. For SB only, quality or duration of sleep assessed using a validated instrument (points on different scales of number of hours of sleep per night).

5. Stress level, assessed using a validated instrument (points on different scales).

6. Quality of life assessed using a validated instrument (points on different scales).

7. Compliance with the intervention (proportion of sessions completed as planned).

8. Nature and duration of any reported side effects.

Outcomes will be segregated by bruxism subtype. If data are available, outcomes will be characterised at the completion of the intervention period and, because we are interested in the potential for long-term changes in bruxism severity, separately at the conclusion of any follow-up after the intervention period. We will segregate outcomes based on the duration of the preceding intervention and follow-up periods. To do this, intervention periods will be divided into three groups: interventions continuing for less than 48 hours; interventions continuing between 48 hours and 7 days; and those continuing for more than 7 days. Follow-up periods will be divided into three periods: less than 3 months, between 3 and 12 months, and 12 months or longer.

By validated instrument we mean any data collection tool that has a recognised standard form for administration and that has been subject to evaluation to demonstrate reliability and construct validity. We recognise that measures of pain and disruption to sleep may reflect beneficial effects of the intervention (reduced pain), side effects (increased or *de novo* pain) or both. Scoping suggests that most research is in the form of laboratory-based pilot studies and that defining implementation-related outcomes like cost would be premature.

### Study types

In respect of review Objective 1 we will include any study type that provides evidence about types of biofeedback, technical aspects of development and any safety-related issues.

For Objective 2 we will include randomised or quasi-randomised controlled trials with end-point comparisons. We will also include randomised cross-over studies but will analyse only the first time period as we expect carry-over to influence subsequent data [38]. For this objective, we will exclude any other study design, namely case series, observational cohorts, case–control and uncontrolled studies, before and after design, case reports, editorials, systematic or other reviews and commentaries. We will also exclude complex interventions where biofeedback forms only one part of treatment as the effects of biofeedback may not be clearly isolated.

### Search methods for identifying studies

The review will be based on a literature search of the electronic databases Cochrane Central Register of Controlled Trials (CENTRAL), MEDLINE, EMBASE, PsycINFO and Web of Knowledge (databases Web of Science^®^, Derwent Innovations Index^SM^ and Chinese Science Citation Database^SM^) using a simple, sensitivity-maximising search strategy (Table 2) tailored to each resource. We will also search clinical trial registries and contact authors of any pending or unpublished studies. We will search the grey literature using OpenGrey and ProQuest Dissertations.

**Table 2:** Generic search strategy

**ID**	**Term**	**Description**
1	brux*	Search for any word with ‘brux’ as its stem.
2	*bruxism/OR *sleep bruxism/	Search for the keywords or subject headings ‘bruxism’ or ‘sleep bruxism’.
3	1 AND 2	

Because of poorly defined and unstandardised terminology, we elected to use a simple, sensitivity- maximising search strategy.

No language or date restrictions will be applied to the search. However, we will only consider articles written in a language other than English if they possess an English abstract. Studies with such abstracts but not available in English will be translated by a commercial translation service. We will review the reference lists of included articles and contact study authors for purposes of clarification or for information on additional relevant published or unpublished studies. Prior to publication, we will perform an update search to identify any new citations.

Two authors (SI and DZ) will perform the search independently and compare the results to ensure accuracy. A third author (KH) will inspect the searches to confirm that they have been carried out correctly. We will keep records of the numbers of articles returned for each term defined for the search separately, and when combined using Boolean operators and before and after removal of duplicates. We will use EndNote X6 (Thomas Reuters, New York, NY USA) software for de-duplication.

### Data collection

#### ***Selection of studies***

After search and removal of duplicates, two authors (SI and DZ) will scan the selected articles to identify any with missing abstracts or titles. These will be located by manually searching the source databases and/or using a general-purpose search engine. Two authors (SI and DZ) will then independently examine titles and abstracts to exclude obviously irrelevant articles. Decisions from this initial screening phase will be compared and any discrepancies about which articles to retain resolved by discussion. One author (DZ) will then obtain full text copies, including translations if required, of all potentially relevant studies. The same authors, working independently, will then assess articles against the full inclusion criteria (Table 3) to confirm that they should be included in the review. Disagreements will be resolved through discussion. If no agreement can be reached, KH will act as arbiter. A log will be kept of all screening and full-text decisions. Reasons for exclusion of articles at the full text stage will be recorded for publication in the review. We will use a PRISMA chart for showing study selection process and reasons for exclusion.

**Table 3:** Inclusion and exclusion criteria

**Inclusion criteria**	**Exclusion criteria**
Adults 18 or older	Children <18
Clinical or EMG/PSG-based diagnosis of SB or clinical diagnosis of AB	Co-existing diagnosis of SB and AB (unlikely)
Co-existing diagnoses not affecting clinical management of bruxism	Known neurological or psychiatric comorbidities and/or a past history of receiving biofeedback therapy for bruxism
Biofeedback as an intervention for reducing or controlling bruxism	Relaxation therapy not linked to bruxism specifically
Any valid comparison (placebo, other intervention or biofeedback type)	Co-existing alternate therapy for bruxism in intervention group (complex intervention)
Any duration	Duration of active therapy not clear (or clarified by authors)
Outcomes reported at completion of active therapy and (optionally) at completion of follow-up	-
Any language	-
Published or unpublished work	-
*In respect of Objective 1 only:*	
Any study type that provides evidence about types of biofeedback, technical aspects of design and clinical use or safety issues.	-
*In respect of Objective 2 only:*	
Randomised or quasi-randomised control trials or randomised cross-over studies	Any other study design

AB, awake bruxism; EMG, electromyograph; PSG, polysomnograph; SB, sleep bruxism.

### Data extraction and management

Two authors, SI and DZ, will independently extract data from included studies using a structured form. We plan to extract relevant data on study (for example, design, setting, country), population (for example, age, gender, type of bruxism, co-morbidity), intervention (for example, type, length), and outcome characteristics (for example, type of measurements, follow-up timing). The data extraction forms completed by each reviewer will be compared and discrepancies followed up with reference to the original article. We will contact authors to obtain missing or incomplete data. A pilot extraction of three studies will be performed and the extraction reviewed by a third author (KH) before continuing. At the completion of data extraction, all extracted numeric data will be manually re-checked against the original source by two authors (KH and DZ), working independently. For additional information on data extraction see Additional file 1.

#### ***Assessment of risk of bias***

For randomised controlled trials, we will use the Cochrane Collaboration’s tool for assessing the risk of bias in randomised controlled trials [39]. Two review authors (SI and DZ), working independently, will assess, for each outcome within each study, the risk of bias as either ‘Low’, ‘High’ or ‘Unclear’ in each of the following domains: random sequence generation, allocation concealment, blinding of participants and personnel, blinding of outcome assessment, incomplete outcome data and selective reporting. Because we are analysing only the first period of any crossover studies, we will use the same method to assess these. For quasi-randomised studies where sequence generation and allocation concealment do not apply, we will use a modified version of the tool that qualitatively compares the baseline characteristics between intervention and control groups. We will not perform risk of bias assessment of commentaries, case reports or descriptive studies included in respect of review Objective 2, assuming that they are at high risk of bias. We will also assess other potential sources of bias including baseline imbalances and strategies for protection against contamination. Initial calibration of risk of bias assessment will be performed by blinded assessment of five studies included in an existing review of bruxism [40]. A third review author (KH) will review the assessments and resolve any disagreements.

### Data analysis

#### ***Measures of treatment effect***

We will examine the characteristics of included intervention studies in order to determine the feasibility of performing a meta-analysis. We will include all the studies that meet our inclusion criteria and report the outcomes of interest. All data will be partitioned for analysis according to the underlying type of bruxism (SB and AB) and nature of comparison (for example, versus placebo, versus other treatment modality). Because we expect a range of different techniques to be used to assess the continuous quantitative outcomes included in the review we will use the standard mean difference as the main measure of treatment effect to both summarise individual study data and pool for analysis. We will report dichotomous data using odds ratios. In the event that some studies report continuous data and some report dichotomised data, we will not collapse continuous outcomes into dichotomous outcomes. Instead, we will report these dichotomised data separately (but not include them in any meta-analysis). We will use a pre-defined significance level of 5% and report values using 95% confidence intervals. We will perform all statistical analyses using Review Manager 5 software (The Nordic Cochrane Centre, Copenhagen: Denmark).

#### ***Unit of analysis***

The unit of analysis will be the patient. For studies where only a proportion of the participants in a group are eligible under our inclusion criteria, we will attempt to obtain data for those participants only.

#### ***Dealing with missing data***

In the case of missing data we will first attempt to obtain the missing information from the study authors. If this is not possible we will perform an analysis of complete cases but assume that the data are high risk of bias.

#### ***Assessment of heterogeneity***

We will use data extraction and tabulation to systematically characterise possible sources of clinical and methodological diversity and will use these characteristics to inform a decision to undertake a pooled meta-analysis for each of the planned subgroups. We will use the I^2^ statistic to assist decision making (using the interpretation scheme provided by the Cochrane collaboration [41]) but have chosen not to set a fixed threshold for meta-analysis.

#### ***Assessment of publication bias***

We will characterise possible sources of reporting bias on a per-study basis during quality assessment. If there are at least 10 studies, we will use a funnel plot to explore possible reporting bias at an aggregate level using visual inspection.

#### ***Subgroups***

A number of factors may influence the efficacy of biofeedback and, recognising this, we have pre-defined subgroups into which data will be segregated, if possible, for analysis of the primary outcome: (1) studies partitioned by type of biofeedback according to the taxonomy generated for the review (review Objective 1; for example, auditory, vibratory, gustatory and CES); (2) studies partitioned by duration of follow-up, grouped into short-, medium- and longer-term follow-up (according to our prespecified cut-offs for these periods); (3) participants with co-existing bruxism-related morbidity (for example, TMD) versus those without; and (4) studies partitioned into industry-funded and non-industry-funded groups.

#### ***Meta-analysis and sensitivity analyses***

If, after review, data demonstrate adequate homogeneity we will generate a pooled effect estimate for each subgroup for each outcome using a random effects meta-analysis (DerSimonian-Laird method [42]). We will estimate an effect pooled across subgroups only if it is meaningful to do so after review of heterogeneity and other quality issues, using a random-effects model.

Sensitivity analyses will be used to assess the robustness of the findings in the case that: (1) one or more studies contributing to the estimate presents a concern because of possible biases, or other quality or conduct issues; (2) one or more crossover trials are included in the synthesis; (3) the form of the included biofeedback devices has substantially changed because of technological advancements (for example, miniaturized wearable devices versus a bedside wired devices) which might affect outcomes like acceptability; and (4) there are more than five studies in the meta-analysis and either one or two studies account for more than 50% of the weight.

### Synthesis

We will use the PRISMA statement to structure the reporting of the systematic review [43]. We will generate an initial narrative synthesis summarizing different types of biofeedback available (review Objective 1) by devising and refining a taxonomy to classify the form and content of the included biofeedback interventions. Two authors (SI and DZ), working independently, will come up with an initial schema and this will be refined through discussion.

For those studies contributing to the outcomes assessment (review Objective 2), we will present a narrative summary of included studies presenting their study design, characteristics of participants, characteristics of the interventions and outcomes. We will segregate the information according to the type of bruxism and type of biofeedback being reported, using the taxonomy developed in the initial synthesis. We will also present a ‘Characteristics of included studies’ table describing the methods, participants, interventions and outcomes of individual intervention studies. We will summarize the quantitative effects for each outcome and the overall level of evidence using a summary of findings table and the GRADE approach [44]. Finally, we will present a narrative account highlighting the similarities and differences identified between the two circadian variants of bruxism (review Objective 3).

## Discussion

No therapy to date has been shown to effectively and permanently cure bruxism [2,8] and most accepted approaches focus mainly on symptom management and prevention of complications [2,8]. Biofeedback therapy holds the potential for inducing long-term changes in behaviour that could include reduction or elimination of symptoms. However, it remains unclear whether biofeedback is an effective method for treatment of bruxism [17]. Consequently, biofeedback has found limited use in routine clinical practice [31]. The results of this systematic review will increase understanding of the scope for biofeedback in the management of bruxism and identify areas where further research is required.

## Abbreviations

AB: awake bruxism; CES: contingent electrical stimulation; EMG: electromyograph; SB: sleep bruxism; PSG: polysomnograph; TMD: temporomandibular joint disorder.

## Competing interests

EC’s post-doctoral research was partially funded by Medotech A/S (now absorbed by Sunstar) and he has been previously involved in several trials about biofeedback treatment of bruxism. EC will not be involved in study selection or data analysis. All other authors declare that they have no competing interests.

## Authors’ contributions

All authors listed have contributed sufficiently to the project to be included as authors, and all those who are qualified to be authors are listed in the author byline. SI: manuscript writing and final approval of manuscript. DZ: manuscript writing and final approval of manuscript. EC: critical feedback on manuscript and final approval of manuscript. JC: conception and design, final approval of manuscript. KH: conception and design; drafted manuscript and final approval of manuscript.

## Supplementary Material

Additional file 1 Data that will be extracted from the studies. Click here for file
