# Supplementary material for: Biofeedback for treatment of awake and sleep bruxism in adults: systematic review protocol
Source: Syst Rev. 2014 May 2;3:42. doi: 10.1186/2046-4053-3-42 (PMC4028105; doi:10.1186/2046-4053-3-42)
Supplement: Additional file 1 — Data that will be extracted from the studies. [file 2046-4053-3-42-S1.docx]

## **Data that will be extracted from studies (where possible)**

| **TYPE OF STUDY** | **DATA TO EXTRACT** |
| --- | --- |
| ALL STUDIES | - Study identifying information - Study methods including: - aims of the study, - study design, - methods of and setting for recruitment, - inclusion and exclusion criteria, - including diagnostic criteria for bruxism, - details of the control and comparison groups, and - incentives for participation - Details of biofeedback intervention: - name of the intervention, - nature of sensing technology, - nature of feedback mechanism, - hardware and software technologies |
| INTERVENTION STUDIES (Randomized control trials (RCTs), non-RCTs and cross-over design studies) | - Description and number of participants (at each stage of the trial) - Geographical setting and place where the intervention was delivered - Details of intervention providers - Outcome details: - time points at which the outcome measurements were taken, - measurement technique, - effect sizes for intervention and control groups and comparison, - any statistical tests performed |
| OTHER STUDY TYPES (Considered for review objective 1, only) | - Implementation or safety issues - Summary of main findings/conclusions |
